# High Proteolytic Resistance of Spider-Derived Inhibitor Cystine Knots

**DOI:** 10.1155/2015/537508

**Published:** 2015-12-30

**Authors:** Kyoko Kikuchi, Mika Sugiura, Tadashi Kimura

**Affiliations:** ^1^Laboratory for Drug Discovery and Glycoscience and Glycotechnology Research Group, Biotechnology Research Institute for Drug Discovery, National Institute of Advanced Industrial Science and Technology (AIST), AIST Tsukuba Central 2, 1-1-1 Umezono, Tsukuba, Ibaraki 305-8568, Japan; ^2^United Graduate School of Drug Discovery and Medical Information Sciences, Gifu University, 1-1 Yanagido, Gifu, Gifu Prefecture 501-1193, Japan

## Abstract

Proteolytic stability in gastrointestinal tract and blood plasma is the major obstacle for oral peptide drug development. Inhibitor cystine knots (ICKs) are linear cystine knot peptides which have multifunctional properties and could become promising drug scaffolds. ProTx-I, ProTx-II, GTx1-15, and GsMTx-4 were spider-derived ICKs and incubated with pepsin, trypsin, chymotrypsin, and elastase in physiological conditions to find that all tested peptides were resistant to pepsin, and ProTx-II, GsMTx-4, and GTx1-15 showed resistance to all tested proteases. Also, no ProTx-II degradation was observed in rat blood plasma for 24 hours* in vitro* and ProTx-II concentration in circulation decreased to half in 40 min, indicating absolute stability in plasma and fast clearance from the system. So far, linear peptides are generally thought to be unsuitable* in vivo*, but all tested ICKs were not degraded by pepsin and stomach could be selected for the alternative site of drug absorption for fast onset of the drug action. Since spider ICKs are selective inhibitors of various ion channels which are related to the pathology of many diseases, engineered ICKs will make a novel class of peptide medicines which can treat variety of bothering symptoms.

## 1. Introduction 

Proteolytic degradation in gastrointestinal (GI) tract and blood plasma is a major barrier for peroral peptide drug development [1–4]. So far, despite high target selectivity, peptides are long thought to be unsuitable for oral delivery due to their susceptibility to degradation and low bioavailability. At this time, more than 60 FDA-approved peptide medicines are on the market, approximately 140 peptide drugs are currently in clinical trials, and more than 500 therapeutic peptides are in preclinical stage [5]. *ω*-Conotoxin MVIIA, known as ziconotide or Prialt, is the first FDA-approved peptide drug from marine cone snail* Conus magus* to treat severe chronic pain; however, ziconotide needs to be intrathecally administered because of poor* in vivo* stability [6]. Thus, many peptides are susceptible to enzymatic degradation and most peptide drugs are administered by the parenteral route and approximately 75% are given as injectables today [5]. Nevertheless, noninvasive methods are usually preferred besides life-threatening situation, and variety of noninvasive delivery methods, such as buccal, nasal, and transdermal routes, are being developed since small size of ICK itself has an advantage in absorption [7]. Even so, oral route is the most familiar form of drug delivery in everyday-life. Several strategies to enhance proteolytic stability and bioavailability have been developed for oral administration of therapeutic peptides [8]. Modification of peptide termini, replacement of labile amino acid, and cyclization of a peptide are used to promote peptide stability in GI tract and plasma [7]; increasing molecular mass by PEGylation, coadministration of enzyme inhibitor and permeation enhancers, and encapsulation of peptides in biodegradable polymer microspheres and liposomes are employed to improve plasma half-life of therapeutic peptides [4]. Clark et al. [9] demonstrated that backbone cyclization of conotoxin improved the proteolytic stability while maintaining biological activity, and fully bioactive *α*-selenoconotoxins were synthesized from *α*-conotoxin ImI by replacing cysteine residues with selenocysteine by Armishaw et al. [10].

Cystine knot peptides are usually composed of approximately 30 amino acids having characteristic sturdy structure consistent with 3 antiparallel *β*-sheets “knotted” by 3 disulfide bonds between 6 cysteine residues. This structure is common to most cystine knot peptides and endows them with unusual proteolytic, thermal, and chemical stability, thus making cystine knot peptide promising scaffolds for new peptide drugs [11–15]. During the long course of evolution, cystine knot peptides have evolved to possess multifunctional properties in many species by modifying functional loops exposed from rigid cystine knot cores to accommodate their environments; plants have developed protease inhibitors [16] to protect seeds for zoochory; many species have developed antibacterial [17, 18], antifungal [17], and antiplasmodial [19] peptides to prevent infection, and venomous animals have developed ion channel inhibitors to protect themselves and to hunt their prey [6, 18, 20–22].

Inhibitor cystine knots (ICKs), called “knottins,” are linear cystine knot peptides, and, as seen previously, ICKs from spiders, scorpions, and cone snails work as venom toxins and ICKs from plants and arthropods work as antimicrobial peptides [17]. A linear variant of engineered squash cystine knot works as a protease inhibitor and showed a therapeutic potential for inflammatory disorders by blocking must cell tryptase *β* [16]. Insecticidal ICKs from spiders and scorpions [18, 20–22] are widely recognized as neurotoxins that inhibit variety of ion channels and receptors, including K_v_ channels, Na_v_ channels, Ca_v_ channels, Maxi-K calcium-activated K_v_ channels, and NMDA-subtype of glutamate receptors [22]. Ion channels play essential roles in wide range of biological phenomena including neural transduction and muscle contraction [23] and have become fascinating pharmaceutical targets which are related to various diseases such as hypertension, long QT syndrome, diabetes, epilepsy, schizophrenia, depression, and pain [24–26].

To date, excellent stability of ICKs in GI fluid and serum has been empirically recognized among toxicologists as seen in the review by King [8], reporting that ICK peptides alone are exceptionally resistant to proteases, and ICK peptides are stable in human serum for several days and have half-lives in stimulated gastric fluid of >12 h [27]. However, only limited numbers of actual data on ICK degradation have been published. Trypsin inhibitors from soybean, lima bean, and bovine pancreas showed no degradation in stimulated gastric and intestinal fluid [28]; two protease inhibitors from the squash showed resistance to elastase and trypsin [29]; and four ICKs isolated from marine sponge showed excellent proteolytic resistance to pepsin, trypsin, chymotrypsin, and elastase [30].

Herein we report the proteolytic stability of spider-derived ICK peptides in GI tract and plasma enzymes. Four kinds of ICKs, ProTx-I, ProTx-II, GsMTx-4, and GTx1-15, were subjected to degradation by pepsin, trypsin, chymotrypsin, and elastase in physiological conditions. GTx1-15 is a Ca_v_3.1 inhibitor we have cloned and studied for drug development [21]. ProTx-I and ProTx-II are also Ca_v_3.1 inhibitors and used for comparison with GTx1-15 [31, 32]. GsMTx-4 was used as an example of mechanosensitive ion channel [33, 34]. In addition, stability of ProTx-II in rat blood plasma was observed* in vitro*, and ProTx-II concentration in circulation blood was also monitored* in vivo* using LC-MS/MS.

## 2. Materials and Methods

### 2.1. Peptides, Enzymes, and Chemicals

Human prolactin-releasing peptide (hPRP), ProTx-I, ProTx-II, and GsMTx-4 were purchased from Peptide Institute, Inc. (Osaka, Japan). GTx1-15 was obtained from Alomone Labs (Jerusalem, Israel). Pepsin, elastase, glycine, HCl, and Tris-HCl were purchased from Wako (Osaka, Japan). Trypsin and *α*-chymotrypsin were purchased from Sigma-Aldrich (St. Louis, MO, USA) and Tokyo Kasei (Tokyo, Japan), respectively. All enzymes and peptides were dissolved in distilled water to make 100 ng/*μ*L solutions. Glycine was dissolved to make 1 M solution and adjusted to pH2.0 with 5 M HCl and 1 M Tris-HCl (pH8.0) was diluted to 500 mM.

### 2.2. Degradation of Peptides by Enzymes

Four kinds of ICK peptides, ProTx-I, ProTx-II, GsMTx-4, and GTx1-15, were used to study degradation by GI proteases. Human prolactin releasing peptide (hPRP) was used to show a non-ICK example which contains evenly spaced multiple enzyme cleavage sites. The amounts of pepsin, trypsin, chymotrypsin, and elastase added to each system were determined by the amount of each enzyme that completely degraded BSA within 1 hr. To mimic gastric degradation of peptides, 10 *μ*L 100 ng/*μ*L pepsin was mixed with 50 *μ*L 100 ng/*μ*L peptides in 1.5 mL tubes containing 20 *μ*L 1 M glycine (pH2.0) and 20 *μ*L distilled water. To study intestinal degradation of peptides, 10 *μ*L 100 ng/*μ*L trypsin was mixed with 50 *μ*L 100 ng/*μ*L peptides in 1.5 mL tubes containing 10 *μ*L 500 mM Tris-HCl (pH8.0) and 30 *μ*L distilled water; 10 *μ*L 100 ng/*μ*L chymotrypsin was mixed with 50 *μ*L 100 ng/*μ*L peptides in 1.5 mL tubes containing 10 *μ*L 500 mM Tris-HCl (pH8.0), 10 *μ*L 100 mM CaCl_2_, and 20 *μ*L distilled water; 10 *μ*L 100 ng/*μ*L elastase was mixed with each 50 *μ*L 100 ng/*μ*L peptide in 1.5 mL tube containing 10 *μ*L 500 mM Tris-HCl (pH8.0), 10 *μ*L 100 mM KCl, and 20 *μ*L distilled water. Mixtures of peptides and enzymes were incubated at 37°C for 1–4 hours, and 10 *μ*L aliquots was set aside every 1 hour and placed on ice. 5 *μ*L tricine sample buffer was immediately added to the aliquots and denatured at 100°C for 5 minutes to stop digestion.

### 2.3. SDS-PAGE and CBB Staining

Denatured samples were separated by SDS-PAGE using SuperSep Ace 15–20% tricine gel (Wako, Osaka, Japan) at 200 V for 1 hour. Coomassie Brilliant Blue staining of the gel was performed with e-Stain 2.0 Protein Staining System using eStain Protein Staining Pad (GenScript, NJ, USA). Detected bands were digitized by an image scanner and quantified with Image Studio Digits (LI-COR Biosciences, NE, USA).

### 2.4. *In Vitro* and* In Vivo* Peptide Degradation in Blood Plasma

#### 2.4.1. *In Vitro* ProTx-II Degradation

To observe peptide degradation* in vitro*, blood was extracted from three 8-week-old male SD rats after anesthesia with isoflurane inhalation and the plasma was separated by centrifugation at 1,850 g for 10 minutes at 4°C. Plasma was stored on ice and used within the day of the experiment. ProTx-II was added to the plasma to make 1 *μ*g/mL mixture and incubated at 37°C for 24 hours, withdrawing 250 *μ*L aliquots at 0, 2, 4, 8, and 24 hours. Samples were kept at −25°C away from light until analysis.

#### 2.4.2. *In Vivo* ProTx-II Clearance from Circulation

To observe ProTx-II concentration in circulation blood* in vivo*, three nonfasted male rats were injected with 0.1 mg/mL/kg of ProTx-II from the femoral vein under isoflurane anesthesia. 450 *μ*L blood samples were taken from the tail vein at 0.083 (5 min), 0.25, 0.5, 1, 2, 4, 8, and 24 hours after the injection using Pasteur pipettes coated with heparin sodium. Plasma was obtained by centrifugation at 10,000 g for 3 minutes at 4°C and stored at −25°C protected from light until analysis. Animal experiments were conducted by Nemoto Science Co., Ltd. (Ibaraki, Japan) in accordance with the guideline of the animal experiment ethics committee (authorization number: 14-0024) and under approval of animal experiment ethics committee of AIST (authorization number: 10150125-A-20131029-001).

### 2.5. LC-MS/MS

For sample preparation, 100 *μ*L plasma was mixed with 20 *μ*L 50% methanol and 200 *μ*L 4% phosphoric acid. Whole sample mixture was added to the Oasis HLB 1 cc/10 mg extraction cartridge (Waters) equilibrated with 1 mL methanol and 1 mL distilled water. The column was washed with 1 mL 5% methanol and eluted with 1 mL methanol. The elute was dried under nitrogen flow and dissolved in 100 *μ*L solvent A/B (30% : 70%, v/v) for LC-MS/MS. 10 *μ*L sample was analyzed by a Waters LC-MS/MS unit; ACQUITY UPLC BEH HILIC, 1.7 *μ*m, 2.1 mm I.D. × 100 mm (Milford, MA) was used at a flow rate of 0.3 mL/min by linear gradient elution (solvent A : solvent B = 30% : 70%, v/v, solvent A: 0.1% TFA, solvent B: acetonitrile) using electrospray ionization for Xevo TQ MS (Waters, MA, USA).

### 2.6. Data Analysis

Measurements were converted to the rate of degradation compared to the undegraded values. Experiments were repeated in triplicate and results are indicated as means ± SEM. Statistical significance was determined by Tukey-Kramer test and *P* values of <0.05 were considered significant.

## 3. Results and Discussion

### 3.1. Spider-Derived ICKs Were Mostly Resistant to GI Proteases

**Figure 1:**
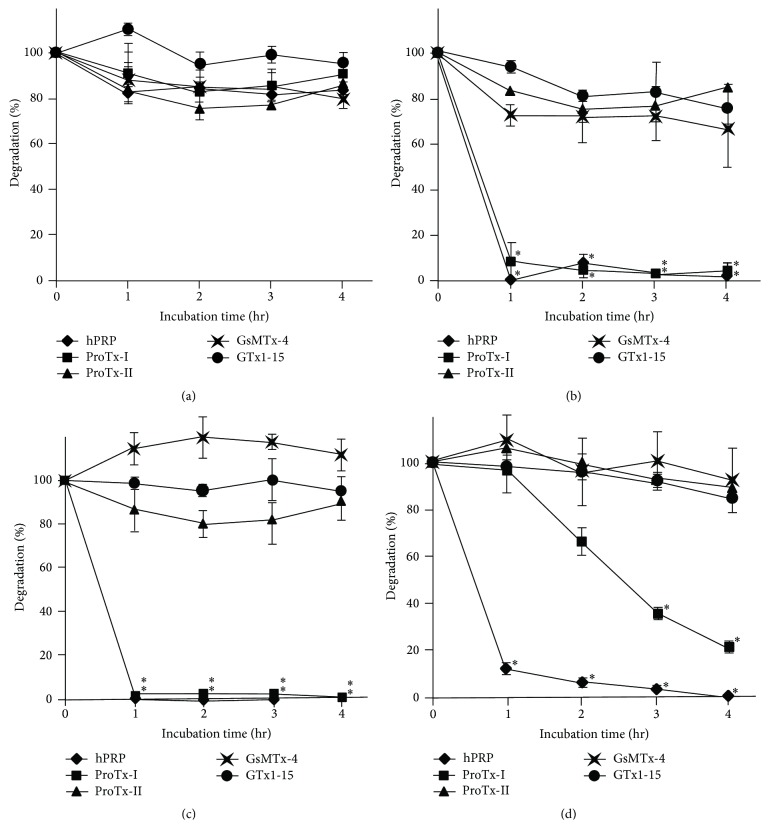
Proteolytic stability of ICK peptides. Four spider-derived ICK peptides and a non-ICK peptide were incubated with representative GI proteases (a) pepsin, (b) trypsin, (c) chymotrypsin, and (d) elastase at physiological conditions for 4 hours. Results are mean ± SEM for 3 experiments. ^*∗*^ Significantly degraded (*P* < 0.05) compared to other undegraded peptides. All peptides were resistant to pepsin, and only ProTx-I was degraded by trypsin, chymotrypsin, and elastase while other ICKs were not degraded.

ProTx-I, ProTx-II, GsMTx-4, GTx1-15, and hPRP were incubated with a gastric enzyme, pepsin, in pH2.0 buffer at 37°C up to 4 hours. In all tested peptides, over 80% of originally added samples remained undigested at the end of the experiment, whereas BSA was completely digested within an hour. GTx1-15 showed exceptional stability and 96% was not degraded (Figure 1(a)). Likewise, the same set of peptides was incubated with intestinal enzymes, trypsin, chymotrypsin, and elastase in pH8.0 buffer at 37°C up to 4 hours. With trypsin, hPRP and ProTx-I were almost completely digested within 1-hour incubation; other ICK peptides were degraded to approximately 80% of the original amount in 2 hours, but the degradation did not proceed beyond that point (Figure 1(b)). Chymotrypsin completely degraded hPRP and ProTx-I within 1 hour; 86% of ProTx-II and 93% of GTx1-15 remained at 4 hours and GsMTx-4 was not degraded at all (Figure 1(c)). Elastase degraded hPRP down to 12% in 1 hour and eventually digested all; ProTx-I was gradually degraded to 22% in 4 hours; ProTx-II, GsMTx-4, and GTx1-15 were also degraded little by little in 4 hours but over 84% remained undigested (Figure 1(d)). Such differential degradation of ICK peptides with proteases was also reported by Werle et al. [29, 36, 37].

ProTx-II, GsMTx-4, and GTx1-15 were hard to degrade in spite of the presence of multiple theoretical protease cleavage sites in their peptide sequences. Alignment of tested ICKs (Figure 3) shows that protease cleavage sites of hard-to-degrade ICKs (GsMTx-4 and GTx1-15) are localized in blocks; the cleavage site distribution of ProTx-I, the only degraded ICK, looks similar to degraded hPRP whose cleavage sites are distributed in scattered manner. These differences in the cleavage site distribution could be related to the differential sensitivity to proteases, and cleavage sites could have been shielded from the binding of proteases by the solid folding of cystine knot structure.

Unlike typical cystine knot peptides, spider venom toxins typically have only 2 antiparallel *β*-sheets [27], which seem to have less protection from proteases (Figure 4). Although ProTx-II has only one short *β*-sheet [35], ProTx-II showed high proteolytic resistance as good as GsMTx-4 and GTx1-15. According to Colgrave and Craik [11], the removal of a single disulfide bond undermined chemical and enzymatic stability while the three-dimensional structure was not largely affected; Heitz et al. [13] also reported that the cystine knot itself seems to be the main factor responsible for the high stability and the cyclization is a stabilizing factor in strongly denaturing conditions. Both their results suggest that disulfide bonds are mainly responsible for the remarkable robustness of the cyclic cystine knot motif rather than cyclization.

### 3.2. ProTx-II Was Not Degraded in Plasma and Showed Fast Clearance from the Circulation

**Figure 2:**
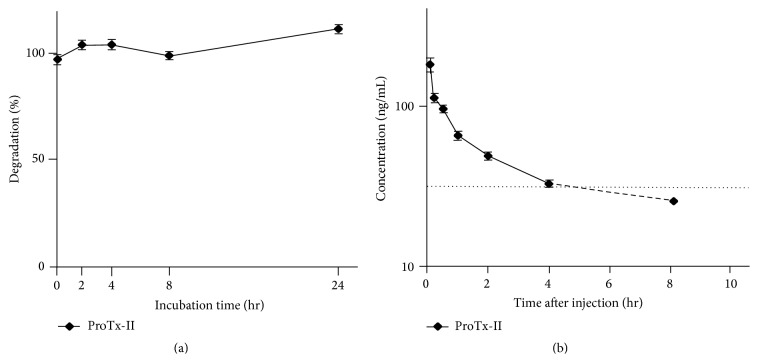
ProTx-II stability in rat plasma. ProTx-II degradation in rat plasma was accessed* in vitro* and* in vivo*. Results are mean ± SEM for 3 experiments. (a) ProTx-II degradation in rat plasma* in vitro*. No ProTx-II degradation was observed in rat plasma* in vitro* for 24 hours. (b) ProTx-II concentration in rat circulation blood* in vivo*. ProTx-II was rapidly eliminated from the circulation. Plasma half-life was 40 min and ProTx-II concentration dropped below the detection limit in 8 hours.

**Figure 3:**
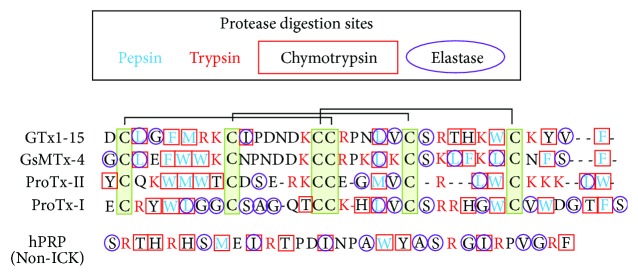
Protease cleavage sites in peptides. Cleavage sites of pepsin, trypsin, chymotrypsin, and elastase are indicated in aligned amino acid sequences. Positions of 6 cystine residues are indicated with green box and 3 disulfide bonds are indicated with connecting lines.

**Figure 4:**
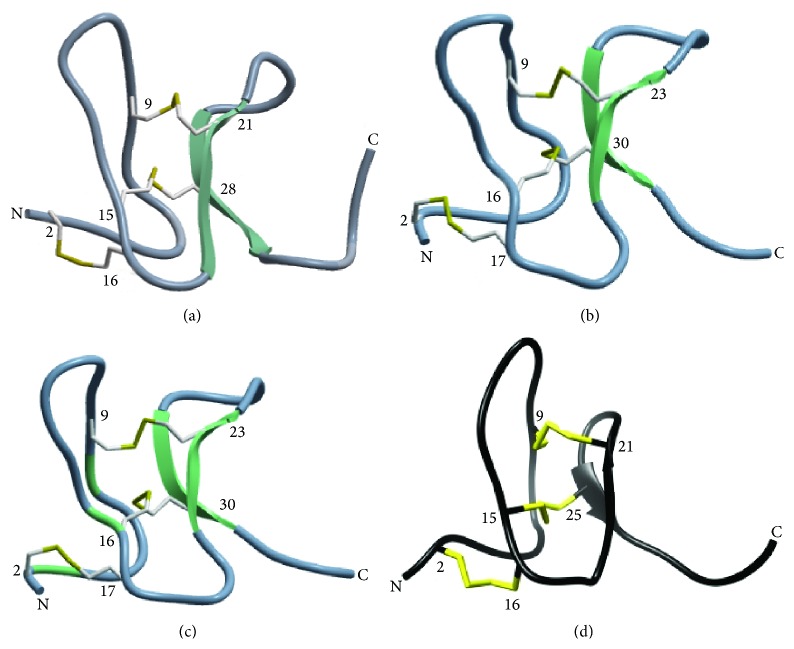
Three-dimensional structures of spider-derived ICK peptides. Protein Data Bank ID numbers for NMR structures of (a) ProTx-I [31] and (b) GsMTx-4 [33] are 2M9L and 1TYK, respectively. 3D structure models of (c) GTx1-15 were constructed by homology modeling with ICM-PRO (Molsoft, La Jolla, CA) based on NMR structures of HnTx-IV (PDB: 1niy). (d) NMR structure of ProTx-II by Park et al. [35]. Reprinted with permission from [35]. Copyright: 2014 American Chemical Society. *β*-Sheets are indicated as green or black arrows and disulfide bonds are highlighted with yellow. Note: spider-derived ICKs have only two antiparallel *β*-sheets and three disulfide bonds except for ProTx-II which has only one *β*-sheet.

ICK degradation in blood plasma* in vitro* and its clearance from circulation* in vivo* were studied with ProTx-II using rats since drug stability in plasma is an integral factor that affects pharmacokinetic parameters such as plasma half-life time of peptides.* In vitro*, 1 *μ*g/mL ProTx-II was mixed with rat blood plasma and incubated at 37°C for 24 hours. ProTx-II was not degraded at all in plasma for 24 hours, indicating that ProTx-II would be absolutely stable once it enters the circulation (Figure 2(a)).* In vivo*, 0.1 mg/mL/kg ProTx-II was administered to rats from the femoral vein to observe ProTx-II concentration in blood over 24 hours to find that within 40 minutes ProTx-II concentration in circulation blood rapidly decreased to half of the concentration immediately after the injection and kept dropping to the detection limit (<30.0 ng/mL) within 8 hours from its administration (Figure 2(b)). Generally, substances with molecular mass below 5 kDa not bound to plasma proteins are excreted via the renal route, which is a main reason for the short peptide half-life time other than enzymatic degradation [4]. Although the affinity of ProTx-II to the plasma protein is unknown at this time, most of ProTx-II was presumed to be cleared by the kidney.

“Short plasma half-life” has been a curse casted on peptide drug development and many attempts were made to prolong plasma half-life. ICK peptides, however, are effective on targets at very low concentration. In case of ProTx-II, even if ProTx-II concentration decreased below detection limit of 30 ng/mL in 8 hr, ProTx-II concentration in circulation is still 7.8 nM, which is sufficient to block Na_v_1.7 whose IC_50_ is 0.3 nM. Although 1 mg/kg dosage of ProTx-II was reported to be lethal in rats, the mortal plasma concentration was 3 *μ*M, which was well above the IC_50_ value for the cardiac sodium channel subtype, Na_v_1.5 and Na_v_1.6 found in central nervous system, and Ranvier nodes [38]. In addition, such fast clearance would be favorable for diagnostic purposes. For example, a spider toxin AgTx was engineered to bind to tumor-associated integrin receptor with high affinity [39].

### 3.3. Stomach Targeted Drugs Could Be Developed from ICKs

Most peroral route drugs are designed to be released in the intestinal tract, and some ICK peptides were shown to permeate through rat intestinal mucosa better than other model drugs [29]. Nevertheless, drugs are constantly exposed to various proteases if the intestine was selected for the site of absorption [40]. In our experiment, all tested ICKs were not degraded by pepsin. Although we need to collect pharmacokinetic data, high resistance for pepsin of these ICKs would enable the development of oral drugs acting at the stomach or absorbed from the stomach. If the stomach were selected for the site of drug release, trypsin is the only formidable protease to handle besides acidic condition [41], and the stomach delivery could become feasible option for ICK-based drugs by combination with permeation enhancers and protease inhibitors. Guggi et al. [42] accomplished stomach targeted delivery of salmon calcitonin by coadministering chitosan-pepstatin A conjugate to prevent degradation by pepsin. Another advantage of stomach targeted delivery is that it takes less time for drugs to reach stomach, which means faster and accurate onset of the drug is possible.

### 3.4. New Functions Can Be Implanted to ICK Scaffolds to Make Better Medicines

As mentioned in the introduction, loop regions of ICK peptides are responsible for multiple functions which come from variety of sequences amenable to directed evolution experiments to find “better” sequences [43–45]. The original function can be optimized and/or new functionalities can be grafted to existing ICK scaffolds and such engineered ICKs are reported to be applied to HIV vaccine [46] and tumor targeting [39, 47]. We have also prepared a random peptide library using GTx1-15 as a scaffold to tune loop sequences with PERISS (intraperiplasm secretion and selection) method, a kind of directed evolution in which a target protein and the interacting peptide are expressed in* E. coli* inner membrane and periplasmic space, respectively [48], and we will soon be able to report what we obtain with GTx1-15 scaffold and its pharmacokinetic data.

## 4. Conclusion

Cystine knot peptides are abundant sources of drug leads thanks to their high target specificity, high affinity, and thermal, chemical, and proteolytic stability. Although our results are preliminary at this time and truly quantitative data should have been presented with LC/MS-MS and NMR, all tested spider ICKs were resistant to pepsin, and ProTx-II, GsMTx-4, and GTx1-15 showed resistance to all tested proteases and most of peptides remained undigested except for ProTx-I which was thoroughly degraded with trypsin and chymotrypsin. No degradation of ProTx-II occurred in plasma for 24 hr* in vitro*; the plasma half-life of ProTx-II* in vivo* was approximately 40 min and the concentration decreased below detection limit in 8 hr. Most of ProTx-II, GsMTx-4, and GTx1-15 were not degraded even though multiple theoretical protease cleavage sites were present, and the cleavage site distribution looked localized in blocks in amino acid sequences. Engineered ICKs will make a novel class of medicines which will take care of annoying pains and symptoms never thought to be healed.

## References

[1] Bernkop-Schnürch A. (1998). The use of inhibitory agents to overcome the enzymatic barrier to perorally administered therapeutic peptides and proteins. *Journal of Controlled Release*.

[2] Choonara B. F., Choonara Y. E., Kumar P., Bijukumar D., du Toit L. C., Pillay V. (2014). A review of advanced oral drug delivery technologies facilitating the protection and absorption of protein and peptide molecules. *Biotechnology Advances*.

[3] Pawar V. K., Meher J. G., Singh Y., Chaurasia M., Reddy B. S., Chourasia M. K. (2014). Targeting of gastrointestinal tract for amended delivery of protein/peptide therapeutics: strategies and industrial perspectives. *Journal of Controlled Release*.

[4] Werle M., Bernkop-Schnürch A. (2006). Strategies to improve plasma half life time of peptide and protein drugs. *Amino Acids*.

[5] Fosgerau K., Hoffmann T. (2015). Peptide therapeutics: current status and future directions. *Drug Discovery Today*.

[6] Schroeder C. I., Craik D. J. (2012). Therapeutic potential of conopeptides. *Future Medicinal Chemistry*.

[7] Truong-Le V., Lovalenti P. M., Abdul-Fattah A. M. (2015). Stabilization challenges and formulation strategies associated with oral biologic drug delivery systems. *Advanced Drug Delivery Reviews*.

[8] King G. F. (2011). Venoms as a platform for human drugs: translating toxins into therapeutics. *Expert Opinion on Biological Therapy*.

[9] Clark R. J., Fischer H., Dempster L. (2005). Engineering stable peptide toxins by means of backbone cyclization: stabilization of the *α*-conotoxin MII. *Proceedings of the National Academy of Sciences of the United States of America*.

[10] Armishaw C. J., Daly N. L., Nevin S. T., Adams D. J., Craik D. J., Alewood P. F. (2006). *α*-selenoconotoxins, a new class of potent *α*7 neuronal nicotinic receptor antagonists. *The Journal of Biological Chemistry*.

[11] Colgrave M. L., Craik D. J. (2004). Thermal, chemical, and enzymatic stability of the cyclotide kalata B1: the importance of the cyclic cystine knot. *Biochemistry*.

[12] Craik D. J., Daly N. L., Waine C. (2001). The cystine knot motif in toxins and implications for drug design. *Toxicon*.

[13] Heitz A., Avrutina O., Le-Nguyen D. (2008). Knottin cyclization: impact on structure and dynamics. *BMC Structural Biology*.

[14] Kolmar H. (2009). Biological diversity and therapeutic potential of natural and engineered cystine knot miniproteins. *Current Opinion in Pharmacology*.

[15] Reinwarth M., Nasu D., Kolmar H., Avrutina O. (2012). Chemical synthesis, backbone cyclization and oxidative folding of cystine-knot peptides—promising scaffolds for applications in drug design. *Molecules*.

[16] Sommerhoff C. P., Avrutina O., Schmoldt H.-U., Gabrijelcic-Geiger D., Diederichsen U., Kolmar H. (2010). Engineered cystine knot miniproteins as potent inhibitors of human mast cell tryptase *β*. *Journal of Molecular Biology*.

[17] Park H. G., Kyung S. S., Lee K. S. (2014). Dual function of a bee (*Apis cerana*) inhibitor cysteine knot peptide that acts as an antifungal peptide and insecticidal venom toxin. *Developmental and Comparative Immunology*.

[18] Gao B., Tian C., Zhu S. (2007). Inducible antibacterial response of scorpion venom gland. *Peptides*.

[19] Pimentel C., Choi S.-J., Chagot B., Guette C., Camadro J.-M., Darbon H. (2006). Solution structure of PcFK1, a spider peptide active against *Plasmodium falciparum*. *Protein Science*.

[20] Kimura T., Ono S., Kubo T. (2012). Molecular cloning and sequence analysis of the cDNAS encoding toxin-like peptides from the venom glands of tarantula *Grammostola rosea*. *International Journal of Peptides*.

[21] Ono S., Kimura T., Kubo T. (2011). Characterization of voltage-dependent calcium channel blocking peptides from the venom of the tarantula *Grammostola rosea*. *Toxicon*.

[22] Smith J. J., Herzig V., King G. F., Alewood P. F. (2013). The insecticidal potential of venom peptides. *Cellular and Molecular Life Sciences*.

[23] Dworakowska B., Dołowy K. (2000). Ion channels-related diseases. *Acta Biochimica Polonica*.

[24] Conte Camerino D., Tricarico D., Desaphy J.-F. (2007). Ion channel pharmacology. *Neurotherapeutics*.

[25] Cannon S. C. (2007). Physiologic principles underlying ion channelopathies. *Neurotherapeutics*.

[26] Mathie A. (2010). Ion channels as novel therapeutic targets in the treatment of pain. *Journal of Pharmacy and Pharmacology*.

[27] Saez N. J., Senff S., Jensen J. E. (2010). Spider-venom peptides as therapeutics. *Toxins*.

[28] Fu T.-J., Abbott U. R., Hatzos C. (2002). Digestibility of food allergens and nonallergenic proteins in simulated gastric fluid and simulated intestinal fluid—a comparative study. *Journal of Agricultural and Food Chemistry*.

[29] Werle M., Schmitz T., Huang H.-L., Wentzel A., Kolmar H., Bernkop-Schnürch A. (2006). The potential of cystine-knot microproteins as novel pharmacophoric scaffolds in oral peptide drug delivery. *Journal of Drug Targeting*.

[30] Li H., Bowling J. J., Su M. (2014). Asteropsins B-D, sponge-derived knottins with potential utility as a novel scaffold for oral peptide drugs. *Biochimica et Biophysica Acta—General Subjects*.

[31] Middleton R. E., Warren V. A., Kraus R. L. (2002). Two tarantula peptides inhibit activation of multiple sodium channels. *Biochemistry*.

[32] Priest B. T., Blumenthal K. M., Smith J. J., Warren V. A., Smith M. M. (2007). ProTx-I and ProTx-II: gating modifiers of voltage-gated sodium channels. *Toxicon*.

[33] Ostrow K. L., Mammoser A., Suchyna T. (2003). cDNA sequence and in vitro folding of GsMTx4, a specific peptide inhibitor of mechanosensitive channels. *Toxicon*.

[34] Suchyna T. M., Johnson J. H., Hamer K. (2000). Identification of a peptide toxin from *Grammostola spatulata* spider venom that blocks cation-selective stretch-activated channels. *Journal of General Physiology*.

[35] Park J. H., Carlin K. P., Wu G. (2014). Studies examining the relationship between the chemical structure of protoxin II and its activity on voltage gated sodium channels. *Journal of Medicinal Chemistry*.

[36] Werle M., Kafedjiiski K., Kolmar H., Bernkop-Schnürch A. (2007). Evaluation and improvement of the properties of the novel cystine-knot microprotein McoEeTI for oral administration. *International Journal of Pharmaceutics*.

[37] Werle M., Kolmar H., Albrecht R., Bernkop-Schnürch A. (2008). Characterisation of the barrier caused by luminally secreted gastro-intestinal proteolytic enzymes for two novel cystine-knot microproteins. *Amino Acids*.

[38] Schmalhofer W. A., Calhoun J., Burrows R. (2008). ProTx-II, a selective inhibitor of NaV1.7 sodium channels, blocks action potential propagation in nociceptors. *Molecular Pharmacology*.

[39] Moore S. J., Leung C. L., Norton H. K., Cochran J. R. (2013). Engineering agatoxin, a cystine-knot peptide from spider venom, as a molecular probe for in vivo tumor imaging. *PLoS ONE*.

[40] Shah R. B., Ahsan F., Khan M. A. (2002). Oral delivery of proteins: progress and prognostication. *Critical Reviews in Therapeutic Drug Carrier Systems*.

[41] Woodley J. F. (1994). Enzymatic barriers for GI peptide and protein delivery. *Critical Reviews in Therapeutic Drug Carrier Systems*.

[42] Guggi D., Krauland A. H., Bernkop-Schnürch A. (2003). Systemic peptide delivery via the stomach: in vivo evaluation of an oral dosage form for salmon calcitonin. *Journal of Controlled Release*.

[43] Kimura R. H., Cheng Z., Gambhir S. S., Cochran J. R. (2009). Engineered knottin peptides: a new class of agents for imaging integrin expression in living subjects. *Cancer Research*.

[44] Silverman A. P., Levin A. M., Lahti J. L., Cochran J. R. (2009). Engineered cystine-knot peptides that bind *α*
_v_
*β*
_3_ integrin with antibody-like affinities. *Journal of Molecular Biology*.

[45] Souriau C., Chiche L., Irving R., Hudson P. (2005). New binding specificities derived from Min-23, a small cystine-stabilized peptidic scaffold. *Biochemistry*.

[46] Stricher F., Huang C.-C., Descours A. (2008). Combinatorial optimization of a CD4-mimetic miniprotein and cocrystal structures with HIV-1 gp120 envelope glycoprotein. *Journal of Molecular Biology*.

[47] Glotzbach B., Reinwarth M., Weber N. (2013). Combinatorial optimization of cystine-knot peptides towards high-affinity inhibitors of human matriptase-1. *PLoS ONE*.

[48] Kubo T., Ono S., Kimura T. (2012). Random peptide library based on spider neurotoxin, and utilization of the library in in vitro evolution derected to GPCR ligands. *Toxicon*.

